# Structural brain morphology in young adult women who have been choked/strangled during sex: A whole‐brain surface morphometry study

**DOI:** 10.1002/brb3.3160

**Published:** 2023-07-17

**Authors:** Jiancheng Hou, Megan E. Huibregtse, Isabella L. Alexander, Lillian M. Klemsz, Tsung‐Chieh Fu, Molly Rosenberg, James Dennis Fortenberry, Debby Herbenick, Keisuke Kawata

**Affiliations:** ^1^ Research Center for Cross‐Straits Cultural Development Fujian Normal University Fuzhou China; ^2^ Department of Kinesiology Indiana University School of Public Health‐Bloomington Bloomington Indiana USA; ^3^ Department of Psychiatry and Behavioral Sciences Emory University School of Medicine Atlanta Georgia USA; ^4^ Department of Applied Health Science, Indiana University School of Public Health Indiana University Bloomington Indiana USA; ^5^ The Center for Sexual Health Promotion, Indiana University School of Public Health Indiana University Bloomington Indiana USA; ^6^ Department of Epidemiology and Biostatistics, Indiana University School of Public Health Indiana University Bloomington Indiana USA; ^7^ Department of Pediatrics, Indiana University School of Medicine Indiana University Indianapolis Indiana USA; ^8^ Program in Neuroscience The College of Arts and Sciences Indiana University Bloomington Indiana USA

**Keywords:** cortical thickness, non‐fatal strangulation, sexual asphyxiation, sexual choking, surface morphometry

## Abstract

**Introduction:**

Being choked/strangled during partnered sex is an emerging sexual behavior, particularly prevalent among young adult women. Using a multiparameter morphometric imaging approach, we aimed to characterize neuroanatomical differences between young adult women (18–30 years old) who were exposed to frequent sexual choking and their choking naïve controls.

**Methods:**

This cross‐sectional study consisted of two groups (choking [≥4 times in the past 30 days] vs. choking‐naïve group). Participants who reported being choked four or more times during sex in the past 30 days were enrolled in the choking group, whereas those without were assigned to the choking naïve group. High‐resolution anatomical magnetic resonance imaging (MRI) data were analyzed using both volumetric features (cortical thickness) and geometric features (fractal dimensionality, gyrification, sulcal depth).

**Results:**

Forty‐one participants (choking *n* = 20; choking‐naïve *n* = 21) contributed to the final analysis. The choking group showed significantly increased cortical thickness across multiple regions (e.g., fusiform, lateral occipital, lingual gyri) compared to the choking‐naïve group. Widespread reductions of the gyrification were observed in the choking group as opposed to the choking‐naïve group. However, there was no group difference in sulcal depth. The fractal dimensionality showed bi‐directional results, where the choking group exhibited increased dimensionality in areas including the postcentral gyrus, insula, and fusiform, whereas decreased dimensionality was observed in the bilateral superior frontal gyrus and pericalcarine cortex.

**Conclusion:**

These data in cortical morphology suggest that sexual choking events may be associated with neuroanatomical alteration. A longitudinal study with multimodal assessment is needed to better understand the temporal ordering of sexual choking and neurological outcomes.

## Graphical abstract

Being choked/strangled during partnered sex is an emerging sexual behavior affecting many young adult women. Our cross‐sectional study using multiparameter brain morphology analysis revealed that being choked/strangled during sex is associated with changes in cortical thickness and other metrics related to altered cortical maturation.

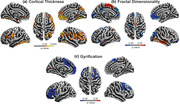

## 1 INTRODUCTION

Adolescents and young adults are in a neurodevelopmental period marked by an exploration of sexual identities, sexual pleasure, and solo and partnered sexual behaviors (Hensel & Fortenberry, 2014). While unintended pregnancy and sexually transmitted infections remain important public health issues, recent studies identified that sexual choking/strangulation or choking a partner during sex, which is a form of manual or ligature strangulation, has become prevalent among adolescents and young adults, disproportionally affecting women (Herbenick, Patterson, et al., 2021; Herbenick, Guerra‐Reyes, et al., 2021; Sun et al., 2017; Wright et al., 2015). In a recent U.S. survey of 4989 college students, 58% of randomly sampled women college students reported having ever been choked during sex, and one‐quarter of these students first experienced being choked during sex between the ages 12 and 17 (Herbenick, Patterson, et al., 2021; Herbenick, Guerra‐Reyes, et al., 2021).

Being choked during sex can induce hypoxic/ischemic stress by restricting the blood flow and air to the brain. When choking ends, the blood flow rushes back to the brain (cerebral reperfusion), and the return of the oxygen is thought to trigger pleasant or euphoric feelings (Herbenick, Fu, et al., 2021). The brain is a resilient organ protected by the blood–brain barrier, capable of neuroplasticity, and equipped with a compensatory network system. However, hypoxemia‐ischemic stress due to choking/strangulation can trigger astrocyte activation to ameliorate neuronal stress by providing erythropoietin (a protein with neuroprotectant properties), secreting vascular endothelial growth factor (VEGF) to maintain cerebrovascular integrity, and reuptaking excess glutamate. These mechanisms of astrocyte activation involve cellular hypertrophy and the proliferation of astrocytes (Sofroniew, 2005) which can lead to morphological changes, such as increased cortical thickness. If hypoxemia‐ischemic stress occurred repetitively over time, it may impact one's mental health and cognition. For example, recurring non‐fatal strangulation in other contexts (e.g., intimate partner violence, the adolescent “Choking Game”) has been shown to contribute to the emergence of depression, post‐traumatic stress disorder, and chronic headaches (Bichard et al., 2021; Busse et al., 2015; Karakurt et al., 2021; Linkletter et al., 2010). Our recent survey revealed that women who had been choked more than five times in their lifetime were twice as likely to report current symptoms related to depression, anxiety, sadness, and loneliness compared to their choking naïve counterparts (Herbenick, Fu, et al., 2021). Altogether, these data suggest that repetitive choking during sex could manifest in an array of physical and emotional symptoms. However, the existing data on sexual choking are extremely limited, and more research is needed to determine the relationship, if any, between sexual choking and neurological outcomes.

The multiparameter morphometric neuroimaging approach using both volumetric features (cortical thickness) and geometric features (fractal dimensionality, gyrification, and sulcal depth) provides a holistic neurological assessment useful in elucidating the neurobiological correlates of sexual choking. The volumetric and geometric features reflect different aspects of biological underpinning; hence, they do not necessarily correlate with one another (Qiu et al., 2014). The cortical thickness measure informs changes in gray and white matter volume, whereas the fractal dimensionality reflects how the white matter surface fits space constraints and is used to investigate brain white matter surface complexity (L. Zhang et al., 2008). The fractal dimensionality is a sensitive metric for detecting white matter changes in normal aging (L. Zhang et al., 2007) and in pathological states, such as multiple sclerosis (Esteban et al., 2007) and epilepsy (Cook et al., 1995). Additionally, the gyrification index represents the level of local cortical folding that relates to the integrality between subcortical and cortex circuits, while the sulcal depth measures the distance between the pial and outer surface, gauging the complicated folding of the cerebral surface. Both gyrification and sulcal depth are informative in gauging normal aging and cortical maturation, as well as neurodegenerative conditions (Ambrosino et al., 2017; Kohli et al., 2019; Madan, 2019).

Our multiparameter imaging technique was incorporated into the current study to explore cortical morphological differences between college‐aged women who reported being frequently choked during sexual events (≥4 times in the past 30 days) and college‐aged women without any lifetime choking experience. Given that there is no study available to establish sound hypotheses, this proof‐of‐concept study aimed to examine the neuroanatomical correlates of sexual choking by addressing the following research questions: (1) what aspects of brain morphology and which brain regions differ between the choking and choking‐naïve control groups and (2) which directions (greater vs. lesser) of the volumetric and geometric features does the choking group exhibit compared to the control group. By addressing these questions, the current study seeks to provide foundational knowledge that is needed to guide future prospective cohort studies.

## 2 MATERIALS AND METHODS

### 2.1 Participants

**FIGURE 1:**
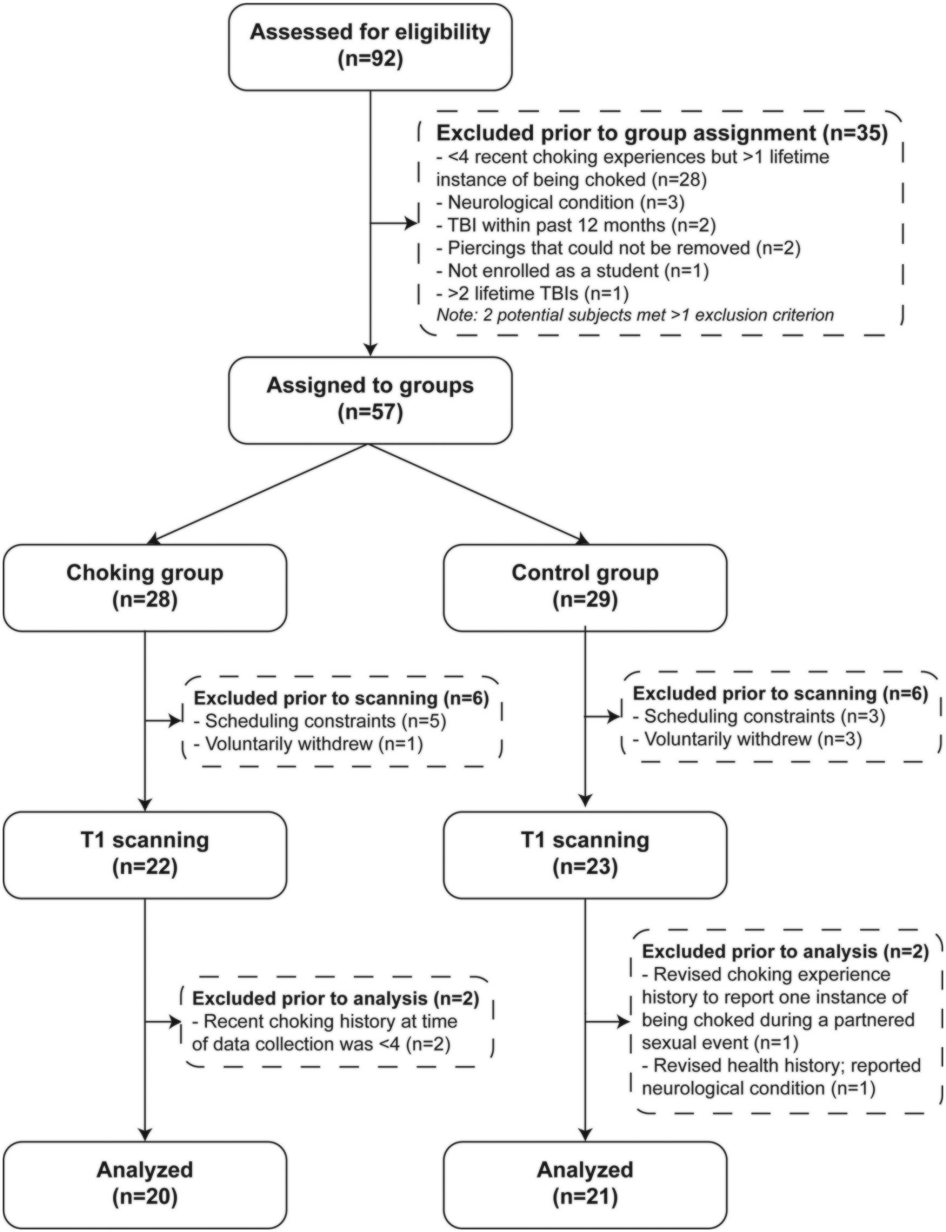
Study flow chart.

This cross‐sectional study consisted of two groups (choking group vs. choking‐naïve group) and was conducted from February 2021 to June 2021 at a large public university. Research participants were recruited via two mechanisms: (1) respondents in a separate university‐wide survey study could indicate that they were interested in being contacted about a study on sexual behaviors and were asked to provide their email addresses, and (2) additional participants were recruited using the university online classifieds section. Following consent to study participation, all subjects completed a screening questionnaire to determine the eligibility and choking experience status. For general inclusion, subjects were required to be birth‐assigned females, enrolled as at least a part‐time student at Indiana University, and between 18 and 30 years old. For the choking group, additional inclusion criteria were that they reported having been choked four or more times during consensual partnered sexual events in the past 30 days, whereas women in the choking‐naïve group needed to have been free of any lifetime experience of being choked during a partnered sexual event. Subjects in both groups were excluded if they were pregnant, had a traumatic brain injury (TBI) within the past year, reported a history of more than two TBIs, had any magnetic resonance imaging contraindications (e.g., metal inside body near neck, face, and head, metal intrauterine device, and severe claustrophobia), or had a neurological condition (e.g., epilepsy, neurodegenerative disease, aneurysm, tumor, and spinal cord injury). A target sample size was pre‐determined based on our magnetic resonance imaging (MRI) data from the previous research in subconcussive neural injury since there was no biobehavioral study available in the realm of sexual choking research. Our morphology data resulted in large effect sizes (d = 1.2–1.8) between athletes with a history of impact exposures and those athletes without. Thus, 20 participants per group were estimated to yield a statistical power of 80% with a significance level of *α* = .05. After confirming eligibility and group assignment, those who qualified for the study were scheduled for data collection (see Figure 1). The Indiana University Institutional Review Board approved the study, and subjects provided written informed consent prior to participation.

### 2.2 Questionnaires

A health history questionnaire was administered to obtain participants’ demographic information and screen for inclusion and exclusion criteria. A questionnaire about sexual choking experience included questions about the frequency of sexual choking in the past 30 days, 60 days, and 12 months, their lifetime frequency of choking, as well as bruising in the neck after sexual choking, and lifetime frequency of loss of consciousness due to sexual choking. Following these questionnaires, participants completed paper versions of the following mental health scales.

Depression‐related symptoms were assessed using the depression module of the patient health questionnaire (PHQ‐9) (Kroenke & Spitzer, 2002). Each of the nine PHQ‐9 depression items describes one symptom corresponding to one of the nine Diagnostic and Statistical Manual of Mental Disorders, Fourth Edition diagnostics. Generalized anxiety disorder was assessed using the Generalized Anxiety Disorder Assessment (GAD‐7) (Spitzer et al., 2006). The Alcohol Use Disorders Identification Test (AUDIT) is a 10‐item screening tool developed by the World Health Organization to assess alcohol consumption, drinking behaviors, and alcohol‐related problems (Bush et al., 1998). When group differences in PHQ‐9, GAD‐7, and AUDIT were observed, they were included in statistical models as confounders.

### 2.3 Magnetic resonance imaging

The MRI data were acquired on a 3T Siemens Prisma MRI scanner (Siemens, Erlangen, Germany) equipped with a 64‐channel head/neck coil. Cortical thickness measures were based on high‐resolution anatomical images (T1 weighted) acquired using 3D MPRAGE pulse sequence with the following parameters: TR/TE = 2400/2.3 ms, TI = 1060 ms, flip angle = 8, matrix = 320 × 320, bandwidth = 210 Hz/pixel, and iPAT = 2, which resulted in 0.8 mm isotropic resolution.

### 2.4 Cortical surface preprocessing

The Computational Anatomy Toolbox (CAT12; http://www.neuro.uni‐jena.de/cat/), which is a plug‐in software based on Statistical Parametric Mapping (SPM12, https://www.fil.ion.ucl.ac.uk/spm/software/spm12/), was used for the T1‐weighted MRI data preprocessing. The preprocessing consisted of bias‐field correction, skull‐stripping, and alignment to the Montreal Neurological Institute structural template to classify gray matter (GM), white matter (WM), and cerebrospinal fluid (CSF). Spatial normalization was conducted with the Diffeomorphic Anatomical Registration Through Exponentiated Lie Algebra registration (1.5 mm) (Li et al., 2021). Subsequently, a spherical harmonic method was used to reparametrize the cortical surface mesh based on an algorithm that reduces area distortions to repair any topological defects (Li et al., 2021; Yotter et al., 2011).

Cortical thickness was analyzed based on the workflow specified in a previous study (Dahnke et al., 2013). A voxel‐based distance method was used to estimate the WM segment by calculating the distance from the inner GM boundary. Values at the outer GM boundary in the WM distance map are projected back to the inner GM boundary to generate the GM thickness. Followed by this, a central surface was created at the 50% level of the percentage position between the WM distance and GM thickness. For the resultant central surface, a topology correction based on spherical harmonics was used to account for topological defects (Li et al., 2021). The central surface was reparameterized into a common coordinate system through spherical mapping (Desikan et al., 2006). The cortical thickness data were spatially smoothed with a Gaussian kernel with a 15 mm full‐width at half‐maximum (FWHM) for this analysis.

The fractal dimensionality estimates cortical fold complexity based on spherical harmonics (Desikan et al., 2006) and is calculated as the slope of a logarithmic plot of surface area versus the maximum value, where the maximum value is a measure of the bandwidth of frequencies used to reconstruct the surface shape (Khundrakpam et al., 2017). Based on the spherical harmonics, the gyrification, as an indicator of cortical folding, was calculated as absolute mean curvature (Luders et al., 2006). Mean curvature is an extrinsic surface measure and provides information about the change in normal direction along the surface. The sulcal depth measures the depth of sulci and is calculated as the Euclidean distance between the central surface and its convex hull based on the spherical harmonics, then transformed with the sqrt function (Luders et al., 2006). A 25 mm FWHM Gaussian kernel was used in the spatial smoothing step for fractal dimensionality, gyrification, and sulcal depth analyses.

### 2.5 Statistical analysis

Demographic differences between the choking and choking‐naïve groups were assessed with *t*‐tests and chi‐square tests. Group comparison of cortical thickness, fractal dimensionality, gyrification, and sulcal depth was performed using CAT12 and analyzed via a non‐parametric permutation technique. The threshold‐free cluster enhancement (TFCE) was used in the permutation test, which gives cluster‐based thresholding for familywise error correction (Qiu et al., 2014). As a result, the TFCE *p*‐value images obtained were fully corrected for multiple comparisons across space. Because of statistically significant group differences in age, race, and AUDIT, these demographic factors were included in the model as covariates. We reported brain regions with a cluster size of at least 100 vertices (cluster size × percentage covered in the specific region produced by CAT12). The Desikan–Killiany atlas (DK40) (Potvin et al., 2017) was used to label the cortical regions, and the results were visualized using the CAT12. *p*‐Values of less than .05 were considered statistically significant.

## 3 RESULTS

### 3.1 Demographic characteristics

A total of 92 participants were screened for eligibility, and 57 participants who met inclusion criteria and were free of exclusion criteria were assigned to either the choking group (*n* = 28) or the choking‐naïve group (*n* = 29). We were unable to obtain MRI data from 12 participants (choking *n* = 6; choking‐naïve *n* = 6) due to claustrophobia and scheduling conflict, and two participants were retroactively excluded from each group for either not being free of exclusion criteria or not meeting all inclusion criteria upon reexamination of their questionnaire responses (e.g., participants in the choking group stopped engaging in sexual choking between the consent meeting day and the data collection day, and vice versa for participants in the choking naïve). As a result, a total of 41 participants (choking *n* = 20; choking‐naïve *n* = 21) contributed to the cortical morphometry analysis. See Figure 1 for the study flow.

The participants in the choking group had been choked a median of 7, 15, and 42 times in the last 30 days, 60 days, and 12 months, respectively (Table 1). There were several demographic differences observed between groups. The choking‐naïve group was significantly older than the choking group by 2.2 years, and the choking group reported higher scores for AUDIT compared to that of the choking‐naïve group. The choking group included more racially diverse participants compared to the choking‐naïve group. There were no group differences in depression scores (PHQ‐9) and anxiety scores (GAD‐7).

**TABLE 1:** Demographic characteristics.

Variables	Choking	Choking‐naïve	*p*‐Value
Age, years, mean ± SD	21.1 ± 1.9	23.3 ± 3.1	.009
Race, *n* (%)^a^			.037
White	14 (63)	19 (91)	
Black/African American	4 (18)	0 (0)	
Asian	3 (13)	2 (9)	
American Indian/Alaskan Native	1 (5)	0 (0)	
Ethnicity, *n* (%)			
Non‐Latino/Hispanic	18 (90)	18 (86)	.999
Latino/Hispanic	2 (10)	3 (14)	
No. of lifetime TBI experience, n (%)			.999
Mild	1 (5)	2 (9)	
Moderate/severe	0 (0)	0 (0)	
No. of choking experience, median (IQR)			
Last 30 days	7 (5–12.8)	0	
Last 60 days	15 (10–26.3)	0	
Last 12 months	42 (20–60)	0	
No. of lifetime neck bruise from sexual choking, *n* (%)			
0	17 (85)	21 (100)	
1	3 (15)	0 (0)	
No. of lifetime loss of consciousness from sexual choking, *n* (%)			
0	16 (80)	0 (0)	
1 or more (ranged 1–4)	4 (20)	0 (0)	
Mental health and alcohol use scales, mean ± SD			
PHQ‐9	5.75 ± 4.36	4.14 ± 5.29	.294
GAD‐7	6.25 ± 3.92	4.05 ± 3.72	.073
AUDIT	5.85 ± 4.44	2.85 ± 2.26	.012

Abbreviations: AUDIT, Alcohol Use Disorders Identification Test; GAD‐7, the generalized anxiety disorder assessment; IQR, inter‐quartile range; PHQ‐9, patient health questionnaire‐9 depression scale; TBI, traumatic brain injury.

^a^ Several individuals in the choking group indicated that they identified as more than one race/ethnicity, so the percentages add up to more than 100%.

### 3.2 Group differences in cortical surface measures

#### 3.2.1 Cortical thickness

**FIGURE 2:**
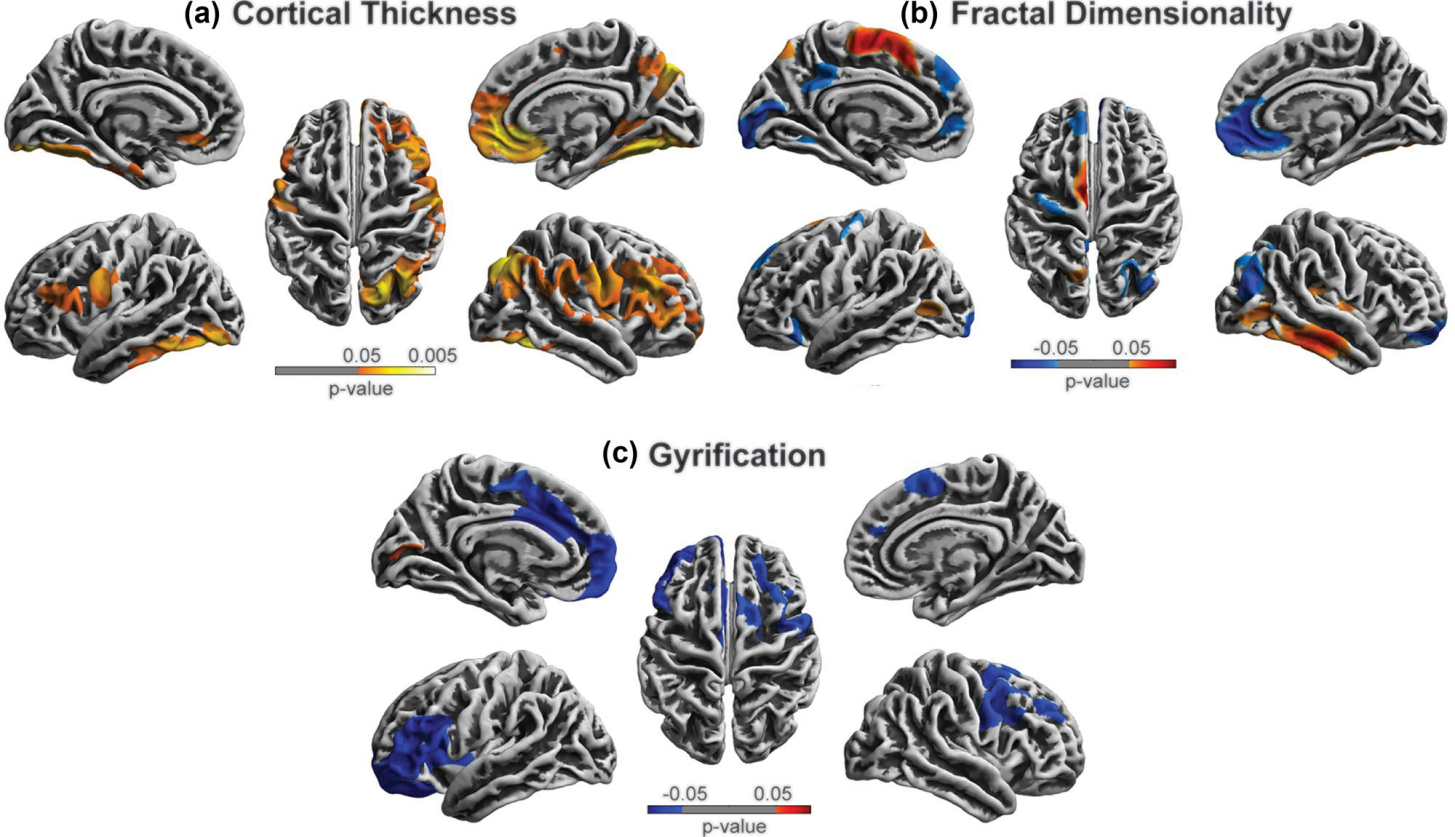
Cortical surface differences between the choking and choking‐naïve groups. The multiple comparison correction was used with non‐parametric permutations (*n*  =  5000) and threshold‐free cluster enhancement (TFCE) correction threshold *p* < .05 after 5000 permutations. Red: the choking group increased compared to the choking‐naïve group. Blue: the choking group decreased compared to the choking‐naïve group.

Compared to the choking‐naïve group, the choking group showed significantly increased cortical thickness in various brain regions. The areas of cortical thickening were observed in the fusiform, lateral occipital, lingual, precentral, postcentral, pars opercularis, rostral middle frontal gyri in both hemispheres, the inferior temporal gyrus in the left hemisphere, the supramarginal, precuneus, superior temporal, pars triangularis, insula, superior frontal, caudal middle frontal, medial orbitofrontal gyri, and inferior and superior parietal lobules in the right hemisphere (see Figure 2a and Table 2).

**TABLE 2:** Differences of cortical surface measures between groups.

Group differences	Brain regions	MNI			Cluster size	*p‐*Value
		*x*	*y*	*z*		
Thickness (left hemisphere)						
Choking > choking‐naïve	Fusiform	−28	−49	−14	489	.010
	Lateral occipital gyrus	−17	−99	−8	334	.010
	Inferior temporal gyrus	−52	−52	−24	322	.010
	Precentral gyrus	−49	3	18	247	.022
	Postcentral gyrus	−53	−3	24	237	.022
	Pars opercularis	55	12	14	197	.033
	Rostral middle frontal gyrus	−36	24	44	184	.033
	Lingual gyrus	−8	−72	−8	141	.010
Thickness (right hemisphere)						
Choking > choking‐naïve	Inferior parietal lobule	44	−64	48	853	.013
	Rostral middle frontal gyrus	8	40	−24	849	.015
	Supramarginal gyrus	54	−30	44	689	.013
	Fusiform	40	−24	−28	662	.005
	Superior parietal lobule	40	−64	52	492	.013
	Medial orbitofrontal gyrus	36	24	44	414	.012
	Precuneus	7	−45	16	361	.013
	Postcentral gyrus	50	−5	34	361	.013
	Pars opercularis	51	12	8	344	.015
	Lingual gyrus	8	−72	−8	337	.005
	Precentral gyrus	43	3	25	328	.013
	Pars triangularis	56	20	13	323	.015
	Superior frontal gyrus	4	32	48	235	.012
	Insula	30	18	7	182	.015
	Lateral occipital gyrus	17	−99	8	175	.005
	Superior temporal gyrus	64	−32	4	131	.013
	Caudal middle frontal gyrus	48	20	−8	121	.015
Fractal dimensionality (left hemisphere)						
Choking > choking‐naïve	Postcentral gyrus	−60	−12	32	427	.003
	Superior parietal lobule	−28	−68	52	107	.028
Choking < choking‐naïve	Lateral occipital gyrus	−48	−68	−2	222	.010
	Superior frontal gyrus	−20	32	48	249	.027
	Pericalcarine	−12	−79	12	208	.010
	Lingual gyrus	−8	−72	−8	172	.010
	Precentral gyrus	−56	0	36	127	.035
	Precuneus	−24	−72	48	123	.019
	Isthmus cingulate cortex	−7	27	29	118	.019
Fractal dimensionality (right hemisphere)						
Choking > choking‐naïve	Middle temporal gyrus	56	−56	−12	340	.007
	Inferior temporal gyrus	60	−48	16	257	.007
	Insula	40	12	−8	235	.030
	Fusiform	40	−24	−28	122	.028
Choking < choking‐naïve	Medial orbitofrontal gyrus	20	28	−20	334	.002
	Inferior parietal lobule	44	−64	48	332	.013
	Lateral orbitofrontal gyrus	20	28	−20	209	.016
	Superior frontal gyrus	4	32	48	154	.002
	Rostral anterior cingulate cortex	4	12	36	128	.002
Gyrification (left hemisphere)						
Choking < choking‐naïve	Rostral middle frontal gyrus	−36	24	44	860	.009
	Superior frontal gyrus	4	31	47	748	.009
	Lateral orbitofrontal gyrus	24	34	−12	486	.009
	Pars opercularis	52	10	5	336	.009
	Pars triangularis	56	20	13	336	.009
	Caudal anterior cingulate cortex	−5	7	37	299	.009
	Insula	30	18	7	224	.009
	Medial orbitofrontal gyrus	−24	34	−12	187	.009
Choking > choking‐naïve	Pericalcarine	−12	−77	12	101	.038
Gyrification (right hemisphere)						
Choking < choking‐naïve	Superior frontal gyrus	4	30	46	459	.028
	Caudal middle frontal gyrus	42	44	14	369	.023
	Precentral gyrus	−52	0	8	347	.023
	Rostral middle frontal gyrus	−48	20	‐8	260	.023

*Note*: The multiple comparison correction was used with non‐parametric permutations (*n*  =  5000) and threshold‐free cluster enhancement (TFCE) correction threshold *p* < .05 after 5000 permutations.

Abbreviation: MNI, Montreal Neurological Institute.

#### 3.2.2 Fractal dimensionality

The fractal dimensionality analysis showed bi‐directional results, such that the choking group exhibited significant increases in fractal dimensionality relative to the choking‐naïve group in the postcentral gyrus and superior parietal lobule in the left hemisphere and in the insula, fusiform, and middle and inferior temporal gyri in the right hemisphere. Conversely, significant decreases in fractal dimensionality were observed in the choking group compared to the choking‐naïve group in the bilateral superior frontal gyrus, the lateral occipital, pericalcarine, lingual, precuneus, precentral gyri, and isthmus cingulate cortex in the left hemisphere, and the medial and lateral orbitofrontal gyri, rostral anterior cingulate cortex, and inferior parietal lobule in the right hemisphere (see Figure 2b and Table 2).

#### 3.2.3 Gyrification index

The choking group showed a significant reduction in gyrification, compared to the choking‐naïve group, in the bilateral rostral middle frontal and superior frontal gyri, the medial and lateral orbitofrontal, pars opercularis, pars triangularis, insula gyri, caudal anterior cingulate cortex in the left hemisphere, and the caudal middle frontal and precentral gyri in the right hemisphere. One region, the pericalcarine in the left hemisphere, showed a small but significant increase in gyrification in the choking group compared to that of the choking‐naïve group (see Figure 2c and Table 2).

#### 3.2.4 Sulcal depth

There were no significant differences in sulcal depth between the groups.

## 4 DISCUSSION

To our knowledge, this is the first study to examine the neuroanatomical characteristics in the brains of young adult women who experienced frequent, recent choking during partnered sexual activities relative to their choking naïve counterparts. There were three primary findings. First, compared to the choking‐naïve group, the choking group had significantly increased cortical thickness in both hemispheres, including the frontal, temporal, parietal, and occipital lobes. Second, the choking group showed increased cortical surface dimensionality in some regions (e.g., precentral gyrus, temporal gyrus) yet decreased dimensionality in many cortical regions, compared to those of the choking‐naïve group. Third, the choking group showed significantly reduced degrees of cortical folding (gyrification) in widespread regions compared to the choking‐naïve group, whereas the depth of the cortical sulci appeared to be similar between the groups. In conjunction with our recently published work on patterns of functional MRI activation during working memory tasks in women with recent, frequent exposure to choking (Huibregtse et al., 2022), these data provide insight into the potential neurobiological consequences of frequent choking/strangulation during sex.

It was unexpected to observe such robust, widespread cortical thickening in the choking group. The direction of changes (thickening vs. thinning) often depends on the brain regions and the nature of the neurologic insults or conditions, such that an instance of cortical thickening does not necessarily indicate a healthier brain than cortical thinning (Fischl & Dale, 2000). For example, patients with autism spectrum disorders and migraine exhibit cortical thickening in many regions of the brain compared to healthy controls (Gaist et al., 2018; Khundrakpam et al., 2017), whereas retired athletes exposed to years of head impacts show cortical thinning compared to controls (Koerte et al., 2016; Wei et al., 2020). In our choking group, we observed significant increases in cortical thickness in the areas that are important for visual processing (e.g., parietal lobule and lateral occipital gyrus), working memory (e.g., middle frontal gyrus), language (e.g., supramarginal gyrus), object recognition (e.g., fusiform gyrus), and motor control (e.g., precentral gyrus). It is possible that individuals who were predisposed to mental illnesses such as depression may already have an altered cortical morphology, and thus, they may become more prone to risky behavior. This explanation is plausible yet unlikely due to the following reasons. First, a recent meta‐analysis concluded that patients with major depressive disorder have been shown to exhibit both cortical thickening and thinning depending on cortical regions (Suh et al., 2019). Second, sensation seeking and the tendency to engage in risky behavior are associated with reduced cortical thickness in adults (Miglin et al., 2019), which was corroborated in a large adolescent cohort (*n* = 427) that impulsive choice was strongly associated with reduced cortical thickness in various brain regions (e.g., ventromedial prefrontal cortex, temporal pole, superior frontal cortex) (Pehlivanova et al., 2018; Schilling et al., 2013). It is important to reiterate that we did not observe any significant group difference in depression and anxiety scores and that we observed increased cortical thickness, instead of decrease, supporting the linkage between sexual choking and cortical morphological changes.

There are various reasons that can increase cortical thickness, ranging from the natural aging process during brain development to genetic influences such as apolipoprotein E ε4 (Espeseth et al., 2008) and presenilin‐1 mutation (Fortea et al., 2010). In the context of sexual choking, hypoxemia/ischemia‐induced reactive gliosis or astrocyte activation may be one of the main reasons for increased cortical thickness. Intermittent and frequent compression of blood vessels in the neck and blockage of the airway during sex can activate astrocytes to (1) protect neurons by providing erythropoietin (a protein with neuroprotectant properties) and also modulating extracellular adenosine levels, (2) secrete VEGF to maintain cerebrovascular integrity, and (3) reuptake excess glutamate in the extracellular space. These mechanisms of astrocyte activation involve cellular hypertrophy, which includes the proliferation of astrocytes (Sofroniew, 2005). This is also noted in our blood biomarker data in that S100B, which is an astrocyte‐enriched protein playing a role in buffering intracellular calcium levels, was significantly elevated in the choking group, compared to the control group, with very good accuracy in distinguishing the groups (Area under the curve = 0.811) (Alexander et al., 2021). Overexpression of S100B is the hallmark molecular mechanism of astrocyte activation, supporting the notion that repetitive choking/strangulation during sex can be cumulative and manifest chronic activation of astrocytes. Although this is a possible mechanism for the cortical thickening in the choking group, it is important to connect the neurobiological data with neurocognitive and neuropsychological function to enhance the clinical implications of our findings. Future longitudinal studies are needed to isolate the temporal ordering of the relationship between sexual choking and short‐ and long‐term neurologic outcomes.

One intriguing aspect of the current study is that increases in cortical thickness in the choking group appear to be more evident in the right than in the left hemisphere. Clinical implication of the right‐lateralization in cortical thickening will require comprehensive cognitive and functional assessments. Yet, our recent fMRI data may provide some insights. In the same cohort of women with or without sexual choking experience, there was a notable inter‐hemispheric imbalance in neuronal activation pattern as detected by the amplitude of low‐frequency fluctuation (ALFF) and regional homogeneity (ReHo). Significantly higher ALFF (↑density of neuronal signal) and ReHo (↑ coherence of neuronal signal) were observed in the right cortical regions in the choking group, whereas lower ALFF and ReHo in the left cortical regions compared to those of the control group (Hou et al., 2023). Although there are no clear agreements between the brain regions showing cortical thickening and increased neuronal activation pattern, it is evident that hemispheric asymmetry was pronounced in the choking group not only in the fMRI metrics but also in the cortical thickness.

While the formation of cortical gyri and sulci takes place during the prenatal period, they continue to enlarge and mature after birth. Gyrification, especially in complex neural network regions such as the prefrontal cortex and precentral and postcentral gyri, continues to mature throughout adolescence and young adulthood (White et al., 2010). Unlike the cortical thickness metric, the direction of change in the gyrification is relatively consistent across several neurologic and psychiatric conditions (e.g., Alzheimer's disease, amyotrophic lateral sclerosis, schizophrenia), where a reduction is associated with negative outcomes, while an increase or maintenance is related to a healthy state (Madre et al., 2020; Ruiz de Miras et al., 2017; Wang et al., 2016; Y. Zhang et al., 2017). In our sample, the group differences in gyrification were found in many cortical regions, but especially in the frontal regions, where structural and functional maturation completes in young adulthood (mid‐20s), including superior and middle frontal gyri, lateral orbitofrontal gyrus, precentral gyrus, and anterior cingulate cortex. All subjects in our choking group had reported experiencing choking during sex for at least the past 12 months. These data on the potential interaction between choking/strangulation during sex and cortical maturation suggest that the neurologic ramifications of being choked during sex could compound over time if exposure is sustained during cortical maturation. However, it is important to note that while gyrification has reflected the potential choking effect, sulcal depth was comparable between the groups. This is perhaps because changes in sulcal depth are more related to age‐related degeneration (Rettmann et al., 2006), and thus no notable changes in these young adult study cohorts.

While sexual strangulation/choking and ischemic stroke are different in severity and duration of stress, evidence from stroke research is helpful when discussing the role of selective vulnerability in certain brain regions to ischemic‐reperfusion stress. Brain ischemia can be focal or diffusive caused by a sudden closure of arteries (e.g., choking/strangulation) or gradual reduction in arterial diameter (e.g., atherosclerosis). Brain tissues under ischemic stress stop operating at normal capacity immediately after and suffer necrosis as soon as 5 min after a complete lack of oxygen and glucose supply, relative to 20–40 min in order body parts. Brain areas with a selective vulnerability are in the arterial border zones, which are the areas between the anterior and middle cerebral arteries (DeSai & Hays Shapshak, 2023). For example, Payabvash et al. (2011) tested 90 patients with acute ischemic (within 12 h of onset) with computed tomography perfusion and found that the precentral, middle, and inferior frontal gyri, along with internal structures, such as insular lobe, paracentral lobule, caudate body, and putamen had the highest ischemic vulnerability to hypoperfusion. These brain regions were also affected in our gyrification and cortical thickness results, suggesting the deleterious neurobiological effects of ischemic‐hypoxemia stress from choking/strangulation during sex. The network analysis between cortical morphology and resting‐state fMRI connectivity would be the important next step to uncover the intersection between neurophysiological and morphological impact due to ischemic‐hypoxemia stress from sexual choking.

Unlike cortical thickness and gyrification, fractal dimensionality exhibited bi‐directional changes, where some regions increased dimension (e.g., postcentral gyrus and temporal cortex), while other regions decreased (e.g., lateral occipital gyrus and medial orbitofrontal gyrus). Fractal dimensionality analyzes the complexity of morphological patterns of the cerebral cortex (Khundrakpam et al., 2017), which is complicated by the opposite patterns of increased cortical thickness and reduced gyrification. Further investigations combining functional and diffusion MRI analysis may allow us to provide a more comprehensive picture of the effects of being choked/strangled during partnered sexual activities.

There are several limitations to this study. Our examination of choking was limited to a small sample at a single site tested in a cross‐sectional design, limiting the generalizability of the results. However, this study provides the first empirical dataset that identifies brain morphological differences in two groups of women categorized by their sexual choking exposure. It is important to acknowledge that this study design precludes any causal inference between sexual choking and brain morphological alteration. A longitudinal study is needed to isolate the temporal link between sexual choking and neurologic outcomes. Self‐reported choking behaviors vary in frequency, intensity, and duration, which are subject to recall bias in survey responses. Therefore, we abstain from conducting a correlation analysis between the frequency of choking exposure and variabilities in cortical morphology within the choking group. Furthermore, given the nature of our non‐interventional design, the time since the last choking event was not controlled for; therefore, the potential that acute choking effects might have contaminated the observed results cannot be eliminated. However, our cortical volumetric and geometric analysis measures intrinsic structural parameters; thus, unlike functional or diffusion MRI metrics, changes in these metrics would occur over time, rather than immediately after an insult (Palacios et al., 2013; Wilde et al., 2012). Lastly, the current study did not solicit information related to substance use, except for alcohol use. Long‐term use of drugs including cannabis can alter brain morphology; however, the effects of substance use were seen in the internal brain regions (e.g., cingulate cortex, putamen, thalamus) without indication of changes in cortical surface gray matter (Pando‐Naude et al., 2021). Moreover, frequent marijuana use was associated with thinner cortices in the temporal and frontal regions (Jacobus et al., 2014), which opposes our observation that the choking group exhibited cortical thickening in those regions. Therefore, previous data argue that the role of substance use in contaminating the observed group differences between the choking and choking‐naïve groups seems negligible.

## 5 CONCLUSIONS

Choking/strangulation during sex is a unique and emerging area of sexual behavior research, overlapping with research in mental health and ischemic/traumatic brain injury. Our morphometry technique identified the potential relationships between frequent exposure to choking/strangulation during sex and alterations in cortical morphologies. Increased cortical thickness, reduced gyrification, and alterations in fractal dimensionality collectively provide the first evidence that frequently being choked/strangled during sex is associated with structural changes in the brain and could possibly alter cortical maturation in young adult women. Young adulthood presents a window of sexual exploration, with choking during sex becoming a popular sexual activity in this population. It is important to acknowledge that choking during sex is the most common form of strangulation, and thus our findings have high public health significance. Further clinical investigation is encouraged to clarify the acute and chronic neurological consequences of being choked during sex using multimodal neurologic assessments.

## AUTHOR CONTRIBUTIONS

Megan E. Huibregtse, Tsung‐Chieh Fu, J. Dennis Fortenberry, Debby Herbenick, and Keisuke Kawatan contributed to the conception and design of the study. Megan E. Huibregtse, Isabella L. Alexander, and Lillian M. Klemsz collected the data and contributed to the interpretation of the data. Jiancheng Hou performed the neuroimaging and statistical analyses. Jiancheng Hou and Keisuke Kawata wrote the first draft of the manuscript. Megan E. Huibregtse, Isabella L. Alexander, Lillian M. Klemsz, Tsung‐Chieh Fu, J. Molly Rosenberg, Dennis Fortenberry, Debby Herbenick, and Keisuke Kawatan revised the manuscript critically for important intellectual content. All authors approved the submitted version and agree to be accountable for all aspects of the work.

## CONFLICT OF INTEREST STATEMENT

The authors declare no conflict of interest.

### PEER REVIEW

The peer review history for this article is available at https://publons.com/publon/10.1002/brb3.3160.

## Data Availability

The datasets generated for this study are available upon request. The raw data supporting the conclusions of this article will be made available by the authors, without undue reservation.
